# Catalytic Asymmetric Synthesis of Both Enantiomers of 4‑Substituted 1,4-Dihydropyridines with the Use of Bifunctional Thiourea-Ammonium Salts Bearing Different Counterions

**DOI:** 10.3390/molecules15118305

**Published:** 2010-11-15

**Authors:** Kohzo Yoshida, Tsubasa Inokuma, Kiyosei Takasu, Yoshiji Takemoto

**Affiliations:** Graduate School of Pharmaceutical Sciences, Kyoto University, Yoshida, Sakyo-ku, Kyoto 606-8501, Japan

**Keywords:** bifunctional thiourea-ammonium salt, aminothiourea, Brønsted acid, 1,4-dihydropyridines, Michael addition, α,β-unsaturated aldehydes, β-keto esters

## Abstract

Organoammonium salts composed of a Brønsted acid and an anilinothiourea promoted the Michael addition of β-keto esters and α,β-unsaturated aldehydes in the presence of primary amines to give functionalized 1,4-dihydropyridines enantioselectively. With the use of the different Brønsted acids such as DFA and HBF_4_ with the same bifunctional thiourea, both enantiomers of 4-substituted 1,4-dihydropyridine were synthesized from the same starting materials.

## 1. Introduction

**Figure 1:**
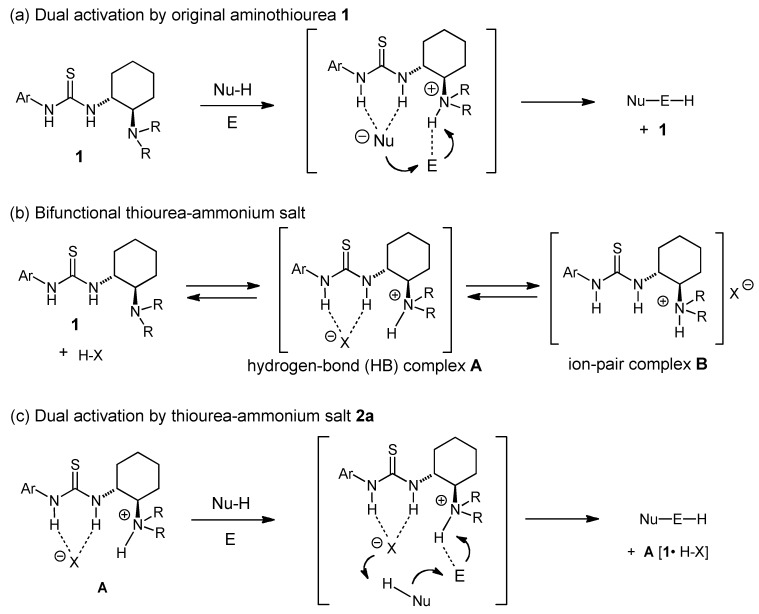
Proposed dual activation mode of aminothiourea.

Asymmetric catalysis using bifunctional catalysts has attracted considerable attention in synthetic organic chemistry. Various types of bifunctional metal- [1,2,3,4] and organo-catalysts [5,6] have been developed and used for catalytic enantioselective reactions over the past decade. Generally, bifunctional acid-base catalysts concurrently activate both nucleophiles and electrophiles to promote addition reactions with high catalytic activity and excellent stereoselectivity via a dual activation mechanism [7,8]. We have previously reported that bifunctional aminothiourea **1** could be used for the asymmetric 1,2- and 1,4-addition of various active methylene compounds to imines and nitroolefins [see (a) in Figure 1)] [9,10,11]. A different approach to asymmetric organocatalysis has been realized through the use of conjugated acid systems, such as in Diels-Alder and aldol reactions [12,13,14,15]. By combining these two concepts, we recently realized Brønsted acid-bifunctional thiourea co-catalysis, in which bifunctional thiourea not only activates an achiral Brønsted acid, but also changes its reaction mode to give the alternative regioisomer as a major product, albeit with moderate enantioselectivity [16]. Our working hypothesis is shown in Figure 1, (b) and (c). When thiourea **1** and a Brønsted acid (HX) are mixed in a 1:1 ratio, ammonium salt complexes **A** and **B** are equilibrated with the starting materials **1** and HX, depending on the acidity of HX and the hydrogen-bonding (H-bonding) ability of the conjugate base (X^-^). If the conjugate base is a strong H-bonding acceptor, H-bonding complex **A**, in which X^-^ is anchored to the thiourea moiety by H-bonds, would be predominant. Otherwise, ion-pair complex **B** might prevail. The difference between the original bifunctional thiourea **1** and ammonium salt **A** is that the conjugate base (X^-^) acts not as a nucleophile, but as a base, which activates a co-existing nucleophile (Nu-H) such as enamino ester or β-keto ester. To explore this hypothesis, a wide range of Brønsted acid-bifunctional thiourea co-catalysts were synthesized and examined. In this article, we describe the details of the versatility of various Brønsted acid-bifunctional thiourea co-catalysts [17,18] together with their application to the asymmetric synthesis of functionalized 1,4-dihydropyridines.

## 2. Results and Discussion

**Scheme 1:**
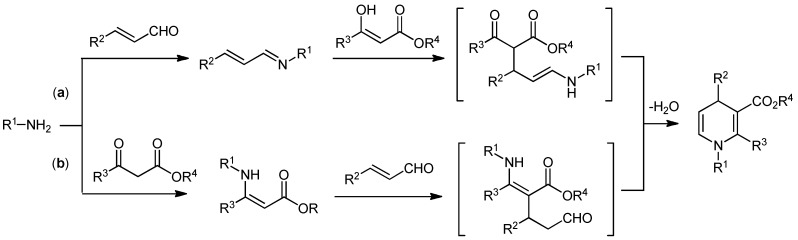
Synthetic routes to 1,4-DHPs.

1,4-Dihydropyridines (1,4-DHPs) and their derivatives are important bioactive compounds and versatile synthetic intermediates in the pharmaceutical industry and in process chemistry. Due to the need for 1,4-DHP derivatives, various synthetic methods have been developed [19,20,21,22]. Although symmetrical 1,4-DHP can be easily prepared by the well-known Hantzsch method [23], new methods for the synthesis of unsymmetrical DHPs are still needed. Furthermore, there have been only a few reports on the organocatalytic enantioselective synthesis of 1,4-DHP [24,25,26]. These routes are shown in Scheme 1. The highly enantioselective synthesis of 1,4-DHP via *route a* from cinnamaldehyde, arylamine, and a 1,3-dicarbonyl compound with a chiral phosphoric acid was achieved by Gong's group [24]. Similarly, Renaud *et al*. reported that another chiral phosphoric acid catalyzed three-component cyclization to afford the product with moderate selectivity (50% ee) via *route b* [25]. Therefore, we examined three-component cyclization via both *routes a* and *b* in the presence of the Brønsted acid-bifunctional thiourea co-catalysts to test their abilities in asymmetric reactions.

### 2.1. Synthesis of chiral bifunctional thioureas 1a-h for Brønsted acid-thiourea co-catalysts

**Figure 2:**
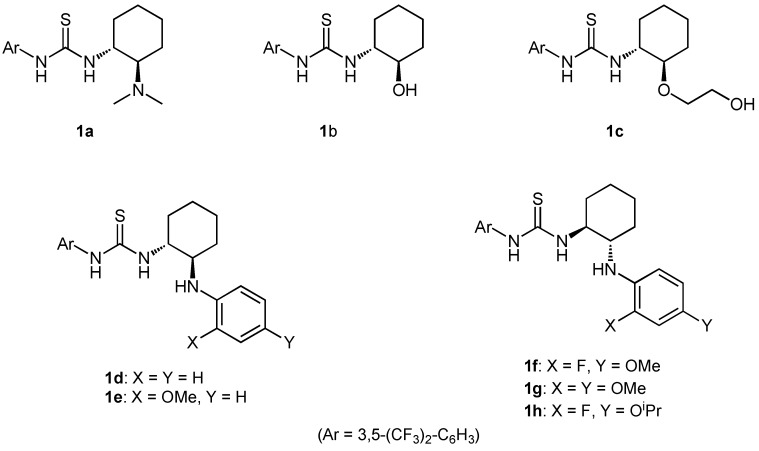
Structures of thiourea catalysts employed.

To investigate the catalytic potential of various Brønsted acid-thiourea co-catalysts, we first synthesized several bifunctional thioureas **1a-h** bearing a functional group, such as a hydroxy or *N*-arylamino group, which have different Brønsted basicities (Figure 2). By changing the basicity of the second functional group of the thiourea catalyst as well as the acidity of Brønsted acid, we can tune both the acidity of the oxonium or ammonium proton and the basicity of the counterion (X^−^).

**Scheme 2:**
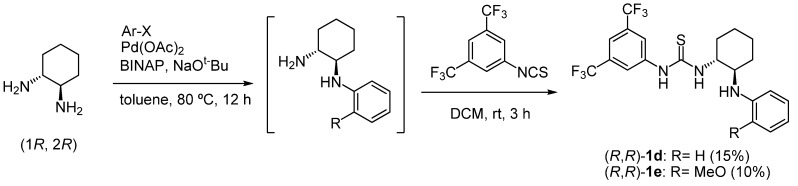
Synthesis of thiourea (*R*, *R*)-**1d **and **1e**.

**Scheme 3:**
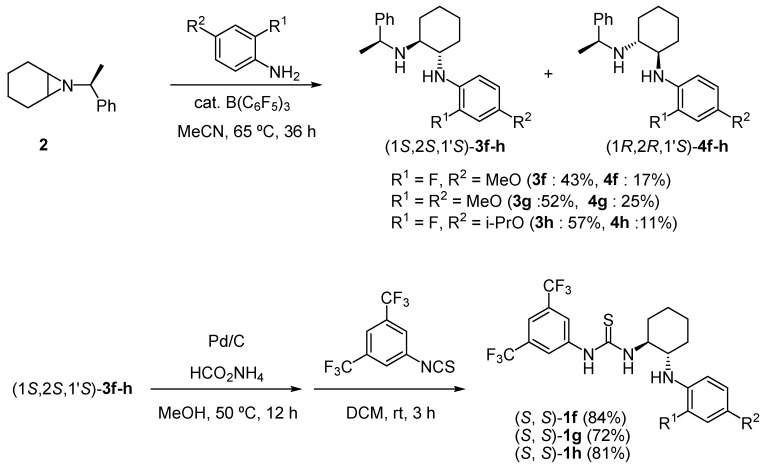
Synthesis of thiourea (*R*, *R*)-**1f-h**.

To synthesize *N*-arylaminothioureas **1d-h**, we examined two synthetic routes. In the first Buchwald-type amination of (*R*,*R*)-1,2-cyclohexyldiamine with appropriate aryl iodides was used as a key step (Scheme 2). However, the key reaction gave the desired products **1d** and **1e** in low yields. We then used the second route to synthesize more functionalized catalysts **1f-h**, which involved the diastereoselective ring-opening of chiral aziridine **2** [27] with functionalized anilines, as shown in Scheme 3. The ring-opening of **2** with the corresponding anilines produced the two diastereomers **3f-h** and **4f-h**. The absolute configurations of **3f** and **4f** were determined to be (1*S*,2*S*,1*S'*) and (1*R*,2*R*,1*S'*), respectively, based on the results of an X-ray single crystallographic analysis of **4f**. The stereochemistries of other products **3g-h** and **4g-h** were deduced from this result for **4f** (Scheme 3). The hydrogenation and thiocarbamoylation of **3f-h** provided the desired thioureas **1f-h** in good yields.

### 2.2. Brønsted acid-bifunctional thiourea co-catalysts for the synthesis of 3,4-disubstituted 1,4-DHPs

We initially investigated the reaction of enamino ester **5a** and α,β-unsaturated aldehyde **6a** in toluene with Brønsted acid-bifunctional thiourea co-catalysts as well as achiral Brønsted acids. Representative results are summarized in Table 1. Notably, strong Brønsted acids such as HBF_4_ and TfOH provided the desired 1,4-DHP **7aa** as a major product, while the same reactions with TFA (trifluoroacetic acid) and DFA (difluoroacetic acid) afforded mixtures of 1,4-DHP **7aa** and 1,2-DHP **8aa** in ratios of 2:1 and 1:2, respectively. In contrast, a weak Brønsted acid such as AcOH did not give any products, and only the starting materials were recovered. These results indicate that the acidity of the catalyst significantly affected the yield and regioselectivity of the products. On the other hand, neither aminothiourea **1a** nor DFA-**1a** co-catalyst furnished any of the desired DHP's in the same reaction. In an attempt to decrease the Brønsted basicity of bifunctional thiourea, we used hydroxythioureas **1b** and **1c** with DFA, but this only had marginal effects on the chemical yield and stereoselectivity. However, the desired product **7aa** was obtained in 74% yield with better regio- and enantioselectivities (**7aa**/**8aa** = 72/17 and 39% ee) with the use of 10 mol% of DFA-chiral *N*-arylaminothiourea **1f** as a co-catalyst.

**Table 1:** Initial screening of various catalysts for the synthesis of 1,4-DHP **7aa**.^a^

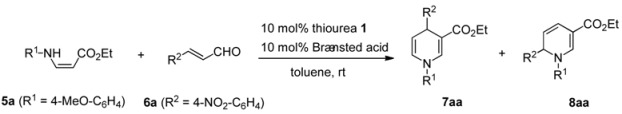									
Entry	Thiourea	BA	Time (h)	Conversion (%) ^b^		7aa		8aa	
				7aa	8aa	Yield (%) ^c^	Ee (%) ^d^	Yield (%) ^c^	Ee (%) ^d^
1	None	HBF_4_	24	40	6		-	-	-
2	None	TfOH	24	40	1	-	-	-	-
3	None	TFA	24	64	35	-	-	-	-
4	None	DFA	24	35	64	-	-	-	-
5	None	AcOH	24	0	0	-	-	-	-
6	(*R*, *R*)-**1a**	None	48	0	0	-	-	-	-
7	(*R*, *R*)-**1a**	DFA	48	0	0	-	-	-	-
8	(*R*, *R*)-**1b**	DFA	36	46	47	33	1	41	1
9	(*R*, *R*)-**1c**	DFA	36	29	56	24	1	48	1
10	(*S*, *S*)-**1f**	DFA	24	79	19	72	39 (*R*)	17	0

^a^ The reactions were carried out with **5a** (0.1 mmol), **6a** (0.1 mmol), thiourea (10 mol%) and Brøsted acid (10 mol%) in toluene (1 mL) at room temperature; ^b^ Conversion as determined by ^1^H-NMR; ^c^ Isolated yield; ^d^ Determined by HPLC.

Since the co-catalysts DFA and *N*-arylaminothiourea **1f **gave good results, a wide range of Brønsted acids were next examined in the presence of **1f** (Table 2). As a result, while the addition of acids [HBF_4_, TfOH, TFA, TCA, perfluorobenzoic acid (PFB)] stronger than DFA (entries 2–6) led to a decrease in enantioselectivity, the concurrent use of **1f** and a weak acid such as AcOH or BzOH significantly improved the enantioselectivity to give the same enantiomer (*R*)-**7aa** with more than 70% ee, albeit in low yield (entries 7 and 8). Since the reaction did not occur with either AcOH or bifunctional thiourea **1f**, we can surmise that bifunctional thiourea **1f** would activate AcOH by forming H-bond complex **A** or ion-pair complex **B**. Since AcO^−^ is well-known to be a good H-bond acceptor, in contrast to BF_4_^−^ and OTf^−^, the H-bond complex **A** could be the actual catalyst. Unfortunately, despite many trials with AcOH and BzOH under various conditions, the chemical yield could not be enhanced without a decrease in ee.

**Table 2:** Effect of Brønsted acids in the presence of **1f** for the synthesis of 1,4-DHP **7aa**.

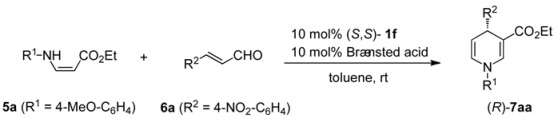				
Entry	Brøsted acid	Time (h)	Yield (%) ^b^	Ee (%) ^c^
1	HBF_4_	48	68	16
2	TfOH	48	78	19
3	TFA	46	64	29
4	TCA	46	83	34
5	DFA	48	72	39
6	C_6_F_5_CO_2_H	24	61	37
7	AcOH	48	11	78
8	BzOH	48	17	75

^a^ The reactions were carried out with **5a** (0.1 mmol), **6a** (0.1 mmol), thiourea (*S*, *S*)-**1f** (10 mol%) and Brøsted acid (10 mol%) in toluene (1 mL) at room temperature; ^b^ Isolated yield. ^c^ Determined by HPLC.

Therefore, we selected DFA as an optimized Brønsted acid and turned our attention to *N*-aryl-aminothioureas **1d-h** to improve the stereoselectivity (Table 3). Due to the instability of enamino ester **5a** under the reaction conditions, the slow addition of **5a** to the reaction mixture of **6a** and co-catalyst **1f**•DFA in toluene was examined, which resulted in the exclusive formation of **4aa** in 86% yield with 50% ee (entry 1). Thus, DFA-catalyzed reactions with several bifunctional thioureas **1e-h** were carried out under slow-addition conditions. The use of phenyl- and mono-substituted anilines **1d** and **1e** as catalysts led to a slight decrease in ee (entries 2 and 3). In contrast, the catalysts **1g** bearing a 2,4-dimethoxyphenyl group gave the same product with a slightly enhanced enantioselectivity, while a similar result was obtained with more bulky catalyst **1h** bearing a 2-fluoro-4-isopropoxyaniline group (entries 4 and 5). Furthermore, other enamino esters **5b** and **5c**, prepared from different primary amines, also underwent cyclization to afford the corresponding products **7ba** and **7ca** with moderate ee's (entries 6 and 7). Despite several trials, we could not improve the enantioselectivity of 3,4-disubstituted 1,4-DHP's **7aa**-**7ca**.

**Table 3:** Effect of *N*-arylaminothioureas in the presence of DFA for the synthesis of 1,4-DHP's.^a^

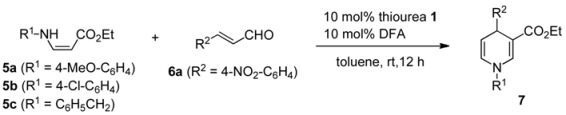					
Entry	Thiourea 1	β-Enamino ester 5	Product 7	Yield (%) ^b^	Ee (%) ^c^
1	(*S*, *S*)-**1f**	**5a**	**7aa**	86	50 (*R*)
2	(*R*, *R*)-**1d**	**5a**	**7aa**	86	42 (*S*)
3	(*R*, *R*)-**1e**	**5a**	**7aa**	47	41 (*S*)
4	(*S*, *S*)-**1g**	**5a**	**7aa**	91	55 (*R*)
5	(*S*, *S*)-**1h**	**5a**	**7aa**	92	50 (*R*)
6	(*S*, *S*)-**1h**	**5b**	**7ba**	78	49 (*R*)
7	(*S*, *S*)-**1h**	**5c**	**7ca**	83	45 (*R*)

^a^ Reaction conditions: Slow addition (0.01 mmol/30 min) of β-enamino esters **5a-c** (0.1 mmol) to a mixture of α,β-unsaturated aldehydes **6a** (0.1 mmol), thiourea **1** (10 mol%) and DFA (10 mol%) in toluene (1 mL) at room temperature. The mixture was stirred for an additional 12 h after completion of the addition; ^b^ Isolated yield; ^c^ Determined by HPLC.

### 2.3. Application of new thiourea-ammonium salts to the synthesis of 2,3,4-trisubstituted 1,4-DHP's

Having succeeded in the catalytic asymmetric synthesis of 3,4-disubstituted 1,4-DHP's, we next applied this method to the asymmetric synthesis of 2,3,4-trisubstituted 1,4-DHP's (Table 4). For this purpose, we first studied the reaction of enamino ester **5d**, derived from ethyl acetoacetate and 4-methoxyaniline, and 3-(4-nitrophenyl)acrylaldehyde **6a** under the optimized conditions using co-catalysts DFA•**1f-h**. In fact, all of the reactions provided the desired product **7da** in 65–93% yields and the highest ee was achieved with DFA•**1h** complex (entries 1-3). Further experiments with β-enamino esters **5e-j** and α,β-unsaturated aldehydes **6a-f** were performed with DFA•**1h** (entries 4-14). With regard to the enamino esters, *tert*-butyl ester **5e** and β-phenyl-substituted analogue **5f** could be used as nucleophiles without a significant decrease in ee (entries 4 and 5). In addition, both electron-rich aryl and arylmethyl groups of **5g-j** could also be tolerated as the substituent (R^1^) on the nitrogen (entries 6 and 12-14). Moreover, the reactions of several unsaturated aldehydes **6b-f** bearing different aryl groups with **5g** provided the corresponding 1,4-DHP's in reasonable yields, but electron-deficient substrates **6d-f** generally led to better enantioselectivity than electron-rich substrates **6b** and **6c** (entries 7–11). The reaction of β-enamino esters with a benzyl group at the nitrogen (R^1^) and a methyl group at the β-position (R^3^) afforded the corresponding 1,4-DHP's **7ia** and **7ja** with good enantioselectivities (entries 13 and 14).

**Table 4:** Scope of the substrates **5** and **6** for the synthesis of 2,3,4,-trisubstituted 1,4-DHP's.^a^

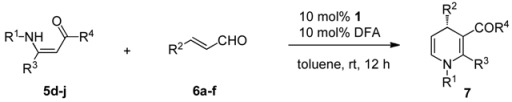										
Entry	Thiourea	5	R^1^	R^3^	R^4^	6	R^2^	7	Yield	Ee
									(%)	(%)
1	(*S*, *S*)-**1f**	**5d**	4-MeO-C_6_H_4_	Me	OEt	**6a**	4-NO_2_-C_6_H_4_	**7da**	84	61
2	(*S*, *S*)-**1g**	**5d**	4-MeO-C_6_H_4_	Me	OEt	**6a**	4-NO_2_-C_6_H_4_	**7da**	65	56
3	(*S*, *S*)-**1h**	**5d**	4-MeO-C_6_H_4_	Me	OEt	**6a**	4-NO_2_-C_6_H_4_	**7da**	93	66
4	(*S*, *S*)-**1h**	**5e**	4-MeO-C_6_H_4_	Me	O^t-^Bu	**6a**	4-NO_2_-C_6_H_4_	**7ea**	81	51
5	(*S*, *S*)-**1h**	**5f**	4-MeO-C_6_H_4_	Ph	OEt	**6a**	4-NO_2_-C_6_H_4_	**7fa**	85	61
6	(*S*, *S*)-**1h**	**5g**	3,4-MeO-C_6_H_3_	Me	OEt	**6a**	4-NO_2_-C_6_H_4_	**7ga**	96	66
7	(*S*, *S*)-**1h**	**5g**	3,4-MeO-C_6_H_3_	Me	OEt	**6b**	C_6_H_5_	**7gb**	61	44
8	(*S*, *S*)-**1h**	**5g**	3,4-MeO-C_6_H_3_	Me	OEt	**6c**	4-MeO-C_6_H_4_	**7gc**	56	38
9	(*S*, *S*)-**1h**	**5g**	3,4-MeO-C_6_H_3_	Me	OEt	**6d**	4-F-C_6_H_4_	**7gd**	62	53
10	(*S*, *S*)-**1h**	**5g**	3,4-MeO-C_6_H_3_	Me	OEt	**6e**	3-F-C_6_H_4_	**7ge**	55	58
11	(*S*, *S*)-**1h**	**5g**	3,4-MeO-C_6_H_3_	Me	OEt	**6f**	2-F-C_6_H_4_	**7gf**	70	44
12	(*S*, *S*)-**1h**	**5h**	4-Cl-C_6_H_4_	Me	OEt	**6a**	4-NO_2_-C_6_H_4_	**7ha**	78	38
13	(*S*, *S*)-**1h**	**5i**	C_6_H_4_CH_2_	Me	OEt	**6a**	4-NO_2_-C_6_H_4_	**7ia**	81	80
14	(*S*, *S*)-**1h**	**5j**	4-MeO C_6_H_3_CH_2_	Me	OEt	**6a**	4-NO_2_-C_6_H_4_	**7ja**	65	77

^a^ Reaction conditions: Slow addition (0.01 mmol/30 min) of β-enaminoesters **5d-j** (0.1 mmol) to a mixture of α,β-unsaturated aldehydes **6a-f** (0.1 mmol), thiourea **1** (10 mol%) and DFA (10 mol%) in toluene (1 mL) at room temperature. The mixture was stirred for an additional 12 h after completion of the addition. ^b^ Isolated yield. ^c^ Determined by HPLC.

### 2.4. Utility of thiourea-ammonium salts derived from strong Brønsted acids and anilinothioureas

We have demonstrated that H-bonding complexes **A**, prepared from anilinothiourea and DFA, efficiently catalyzed the three-component coupling via *route b* to give the functionalized 1,4-DHP's with moderate to good enantioselectivity. We next examined the alternative reaction path via *route a* with the Brønsted acid-anilinothiourea co-catalysts. The reaction was performed as follow. β-Keto ester **10** was added to the preformed imines, prepared from α,β-unsaturated aldehyde **6a** and *p*-anisidine **9**, in the presence of various co-catalysts composed of bifunctional thioureas **1d-h** and Brønsted acids such as DFA, TFA, TfOH, and HBF_4_ (Table 5). Initially we examined the best co-catalyst DFA•(*S*,*S*)-**1h** for route b, which gave the same product (*R*)-**7da** in 64% yield with a slightly low ee (entry 1). Although the same treatment of imine and **10** with co-catalyst TFA•(*S*,*S*)-**1h** led to a similar result, an enantiomer of the product (*S*)-**7da** was obtained, albeit with poor enantioselectivity, with the use of strong Brønsted acids (TfOH, and HBF_4_) as co-catalysts (entries 2–4). The same trend was observed with other bifunctional thioureas (*S*,*S*)-**1f, g** and (*R*,*R*)-**1d**, **e** (entries 5–14). Among the various co-catalysts prepared from **1d-h**, HBF_4_•(*R*,*R*)-**1e** gave (*R*)-**7da** with the highest ee (69% ee) (entry 14). Consequently, we have established a method for the synthesis of both enantiomers of highly functionalized 1,4-DHP's by simply switching the Brønsted acids (DFA and HBF_4_) used as the co-catalysts, starting from the same substrates.

**Table 5:** Three-component cyclization catalyzed by Brønsted acid-anilinothiourea co-catalysts.^a^

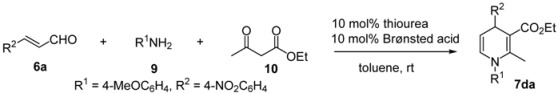					
Entry	Thiourea	Brønsted acid	Time	Yield (%) ^b^	Ee (%) ^c^
1	(*S*, *S*)-**1h**	DFA	48	64	50 (*R*)
2	(*S*, *S*)-**1h**	TFA	24	73	49 (*R*)
3	(*S*, *S*)-**1h**	TfOH	60	82	20 (*S*)
4	(*S*, *S*)-**1h**	HBF_4_	72	69	28 (*S*)
5	(*S*, *S*)-**1f**	DFA	48	53	43 (*R*)
6	(*S*, *S*)-**1f**	TFA	24	76	25 (*R*)
7	(*S*, *S*)-**1f**	TfOH	48	82	50 (*S*)
8	(*S*, *S*)-**1f**	HBF4	72	61	37 (*S*)
9	(*S*, *S*)-**1g**	DFA	48	49	39 (*R*)
10	(*S*, *S*)-**1g**	TFA	24	82	1 (*R*)
11	(*S*, *S*)-**1g**	TfOH	60	77	33 (*S*)
12	(*R*, *R*)-**1d**	TfOH	96	56	39 (*R*)
13	(*R*, *R*)-**1e**	TfOH	96	69	61 (*R*)
14	(*R*, *R*)-**1e**	HBF_4_	72	52	69 (*R*)

^a^ Reaction conditions: The mixture of **6a** (0.15 mmol), *p*-anisidine **9** (0.1 mmol), thiourea **1** (10 mol%) and Brønsted acid (10 mol%) in toluene (1 mL) was stirred at room temperature for 30 min. After keto ester **10** (0.2 mmol) was the added, the resulting mixture was stirred at rt; ^b^ Isolated yield. ^c^ Determined by HPLC.

### 2.5. Proposed reaction mechanism of Brønsted acid-anilinothiourea co-catalysis

**Figure 3:**
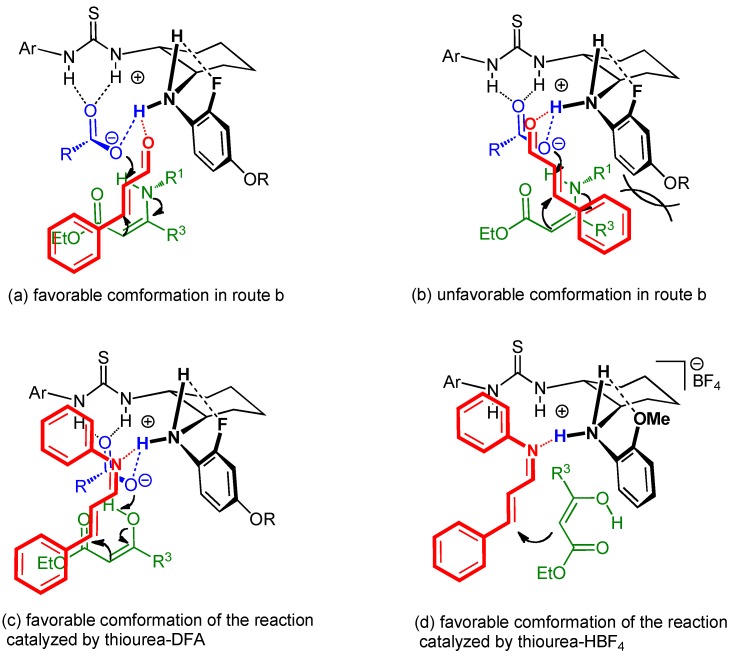
Proposed TS models for the co-catalyzed three-component reaction.

In a former reaction with carboxylic acid-thiourea co-catalysts, H-bonded ammonium complex **A** would be equilibrated with free acid (HX) and uncomplexed thiourea **1** due to the weak acidity of HX (Figure 1). If the free acid can promote the reaction, both the catalyzed and uncatalyzed reactions would proceed, to give the product in low enantioselectivity. This is why DFA, which has medium acidity, gave better results than strong acids such as TFA and TCA. In contrast, high enantioselectivity was achieved with the AcOH•(*S*,*S*)-**1f** co-catalyst, since free AcOH has no catalytic activity for the cyclization. This result obviously indicates that an appropriate bifunctional thiourea can activate weak acids to catalyze three-component cyclization, even though the co-catalysts must be weaker acids than the free acids. To explain this result, we speculate that the conjugate base (X^-^) should play an important role for acceleration of the reaction. Based on this assumption, a proposed reaction mechanism is shown in Figure 3. Initially, the ammonium carboxylate complex **A**, in which each of two ammonium protons interacts with the carboxylate anion or ortho-substituent of the aniline via H-bond, would be formed from the catalyst **1** and HX. The aldehyde would then interact with one of the ammonium protons of the co-catalyst from the less-hindered side. The protonated aldehyde would be attacked from the bottom face (*Si*-face) by the enamino ester, which is concurrently deprotonated by the conjugate base. In this transition-state TS-(a), which is energetically more stable than TS-(b), both nucleophile **5** and electrophile **6** are activated simultaneously by HX complexed with the bifunctional anilinothiourea, to generate the desired (*S*)-product, when (*R*,*R*)-thiourea is used.

Similarly, the reaction of α,β-unsaturated imine and β-keto ester with DFA-(*R*,*R*)-thiourea **1** can be explained by TS-(c) in Figure 3. In this case, (*Z*)-imine [28] should coordinate to the ammonium proton of the same co-catalyst and the nucleophile approaches from the same *Si*-face to predominantly give the (*S*)-isomer. On the other hand, the ion-pair complex **B** would be exclusively generated when strong acids such as TfOH, and HBF_4 _are reacted with bifunctional thiourea **1**. As shown in Figure 3, in TS-(d), the (*Z*)-imine coordinates to the ammonium proton of the ion-pair complex **B** in the same way as in TS-(c), but the nucleophile is considered to approach from the less-hindered upper side (*Re*-face) without any assistance of the conjugate base, since the ammonium proton of the ion-pair complex **B** should be more acidic than that of the H-bonding complex **A**, to predominantly give the (*R*)-isomer.

## 3. Experimental

### 3.1. General

All non-aqueous reactions were carried out under a positive atmosphere of argon in dried glassware unless otherwise noted. Solvents were dried and distilled according to standard protocols. Materials were obtained from commercial suppliers and used without further purification except when otherwise noted. All melting points were determined on a Yamamoto micro melting point apparatus and are uncorrected. ^1^H- and ^13^C-NMR spectra were recorded in CDCl_3_ at 500 or 400 MHz, and at 125 or 100 MHz, respectively; Tetramethylsilane (TMS) was used as an internal standard. IR spectra were recorded on a JASCO FT/IR-410 Fourier-tranfer infrared spectrometer. Low and High resolution mass spectra were obtained by EI or FAB method. Optical rotations were recorded on a JASCO DIP-360 polarimeter with a path length of 1 cm; concentrations are quoted in mg (2 mL). [α]^D^ values are measured in 10^−1^ deg cm^2^ g^−1^. Enantiometeric excess was determined by high performance liquid chromatography (HPLC) analysis. 

### 3.2. Synthesis of 1-(3,5-bis(trifluoromethyl)phenyl)-3-((1R,2R)-2-(2-methoxyphenylamino)cyclohexyl)thiourea *[**(***R***, ***R***)-1e**]*

To a solution of (1*R*,2*R*)-1,2-diaminocyclohexane (251 mg, 2.20 mmol) in toluene (10 mL) was added 2-bromoanisole (374 mg, 2.00 mmol), *rac*-BINAP (124 mg, 0.20 mmol), sodium *t*-butoxide (577 mg, 6.00 mmol) and Pd(OAc)_2_ (22.5 mg, 0.10 mmol). After the mixture was stirred at 80 °C for 12 h and cooled at ambient temperature, the mixture was filtered through a Celite pad. The filtrate was concentrated *in vacuo*, The resulting residue was passed through silica gel pad (hexane-ethyl acetate = 1:1 to CHCl_3_-CH_3_OH- aq.NH_3_ = 100:10:1) to give crude (1*R*,2*R*)-*N*1-(2-methoxyphenyl)cyclohexane-1,2-diamine, which was used in next reactions without further purification. This crude material was dissolved with CH_2_Cl_2_ (5 mL), and added 1-isothiocyanato-3,5-bis(trifluoromethyl)benzene (81.5 mg, 0.30 mmol). After the mixture was stirred at ambient temperature for 1 h, the reaction mixture was concentrated in vacuo. The residue was purified by silica gel chromatography (hexane:ethyl acetate = 5:1) to give thiourea (*R*, *R*)-**1e **(95.1 mg, 0.193 mmol, 10%) as a colorless amorphous solid; IR (ATR) 3326, 2925, 1507, 1091 cm^-1^;^1^H-NMR (500 MHz, DMSO-*d*_6_ at 100 °C) δ (ppm) 9.68 (s, 1H), 8.17 (s, 2H), 7.94 (br, 1H), 7.61 (s, 1H), 6.76 (d, *J* = 8.0 Hz, 1H), 6.75 (dd, *J* = 8.0 and 8.0 Hz, 1H), 6.65 (d, *J* = 8.1 Hz, 1H), 6.51 (dd, *J* = 8.1 and 8.0 Hz, 1H), 4.72 (br, 1H), 4.41-4.38 (m, 1H), 3.71 (s, 3H), 3.33-3.30 (m, 1H), 2.16-2.06 (m, 2H), 1.78-1.64 (m, 2H), 1.49-1.18 (m, 4H); ^13^C-NMR (125 MHz, DMSO-*d*_6_ at 100 °C) δ (ppm) 180.4, 146.4, 141.7, 137.3, 129.8 (q, ^2^*J*_(C, F)_ = 33.8 Hz), 122.8 (q, ^1^*J*_(C, F)_ = 271 Hz), 121.8, 120.7, 115.4, 115.1, 110.4, 109.5, 56.3, 55.9, 55.2, 31.6, 30.7, 23.8, 23.4; MS (FAB^+^) *m/z*: 492 (M + H^+^, 100); HRMS (FAB^+^) *m/z*: calcd for C_22_H_24_F_6_N_3_OS (M + H^+^): 492.1544. Found: 492.1537; [α]_D_^25^ = -6.1 (*c* 1.07, CHCl_3_).

### 3.3. Synthesis of 1-(3,5-bis(trifluoromethyl)phenyl)-3-((1R,2R)-2-(phenylamino)cyclohexyl)thiourea *[**(***R, R***)-1d**]*

A procedure similar to that described for the preparation of **1e **afforded **1d** (15%). Colorless amorphous solid; IR (ATR) 3327, 2932, 2858, 1536, 1091 cm^−1^; ^1^H-NMR (500 MHz, DMSO-*d*_6_) δ (ppm) 9.95 (s, 1H), 8.19 (br, 1H), 8.18 (s, 2H), 7.69 (s, 1H), 7.04 (dd, *J* = 8.1 and 7.5 Hz, 2H), 6.63 (d, *J* = 8.1 Hz, 2H), 6.50 (t, *J* = 7.5 Hz, 1H), 5.36 (d, *J* = 8.6 Hz, 1H), 4.27-4.21 (m, 1H), 3.42-3.29 (m, 1H), 2.33-2.01 (m, 2H), 1.78-1.63 (m, 2H), 1.41-1.13 (m, 4H); ^13^C-NMR (125 MHz, DMSO-*d*_6_) δ (ppm) 179.9, 148.1, 141.8, 130.1 (q, ^2^*J*_(C, F)_ = 33.4 Hz), 128.8, 122.1 (q, ^1^*J*_(C, F)_ = 272 Hz), 121.8, 115.9, 115.6, 112.5, 56.5, 55.1, 31.5, 30.7, 24.1, 23.8; MS (FAB^+^) *m/z*: 462 (M + H^+^, 100); HRMS (FAB^+^) *m/z*: calcd for C_21_H_22_F_6_N_3_S (M + H^+^): 462.1439. Found: 462.1429; [α]_D_^23^ = 34.9 (*c* 1.02, CHCl_3_).

### 3.4. Synthesis of *(1*S*,2*S*,1’*S*)**-3f ***and *(1*R*,2*R*,1’*S*)**-4f***

To a solution of aziridine **2** (603 mg, 3.00 mmol) in CH_3_CN (15 mL) was added 2-fluoro-4-methoxyaniline (430 mg, 3.05 mmol) and tris(perfluorophenyl)borane (154 mg, 0.300 mmol). After the mixture was stirred at 65 °C for 36 h and cooled at ambient temperature, the reaction mixture was added 0.3 g of Amberlyst A-21 resin and 5 mL of dichloromethane. The mixture was stirred for 1 h then the resin was removed by filtration through a cotton plug. The solvent was removed *in vacuo* and the residue was purified by flash amino silica gel chromatography (hexane-ethyl acetate = 5:1) to give (1*S*,2*S*,1’*S*)-**3f** (440 mg, 1.28 mmol, 43%) as a clear oil and (1*R*,2*R*,1’*S*)-**4f** (175 mg, 0.51 mmol, 17%) as a clear oil that solidified to a white solid after standing.

*(1S,2S)-N^1^-(2-Fluoro-4-methoxyphenyl)-N^2^-((S)-1-phenylethyl)cyclohexane-1,2-diamine* [(1*S*,2*S*,1’*S*)-**3f**]: Colorless oil; IR (ATR) 3330, 2979, 2921 cm^−1^; ^1^H-NMR (500 MHz, CDCl_3_) δ (ppm) 7.34-7.19 (m, 5H), 6.75 (dd, *J* = 9.5 and 9.5 Hz, 1H), 6.65 (dd, *J* = 12.5 and 2.5 Hz, 1H), 6.57 (dd, *J* = 9.5 and 2.5 Hz, 1H), 3.89 (q, *J* = 7.5 Hz, 1H), 3.74 (s, 3H), 3.67 (br, 1H), 2.93 (dt, *J* = 3.5 and 9.5 Hz, 1H), 2.48 (dt, *J* = 3.5 and 9.9 Hz, 1H), 2.14-2.09 (m, 1H), 1.89-1.83 (m, 1H), 1.68-1.57 (m, 2H), 1.32 (d, *J* = 7.5 Hz, 3H), 1.29-1.00 (m, 4H); ^13^C-NMR (125 MHz, CDCl_3_) δ (ppm) 152.8 (d, ^1^*J*_(C, F)_ = 242 Hz), 151.9 (d, ^3^*J*_(C, F)_ = 6.0 Hz), 147.3, 130.5 (d, ^2^*J*_(C, F)_ = 12.0 Hz), 128.3, 126.6, 126.5, 115.2 (d, ^3^*J*_(C, F)_ = 3.6 Hz), 109.4 (d, ^4^*J*_(C, F)_ = 3.5 Hz), 102.3 (d, ^2^*J*_(C, F)_ = 22.8 Hz), 60.5, 59.0, 56.2, 55.9, 32.72, 32.66, 24.79, 24.76, 24.1; MS (FAB^+^) *m/z*: 342 (M^ +^, 100); HRMS (FAB^+^) *m/z*: calcd for C_21_H_27_FN_2_O (M^ +^): 342.2107. Found: 342.2110; [α]_D_^26^ = 34.5 (*c* 1.16, CHCl_3_).

*(1R,2R)-N^1^-(2-Fluoro-4-methoxyphenyl)-N^2^-((S)-1-phenylethyl)cyclohexane-1,2-diamine* [(1*R*,2*R*,1’*S*)-**4f**]: white solid; Mp. 57–58 °C (hexane); IR (ATR) 3359, 2973, 2924 cm^−1^; ^1^H NMR (500 MHz, CDCl_3_) δ (ppm) 7.38-7.24 (m, 5H), 6.71 (dd, *J* = 9.2 and 9.2 Hz, 1H), 6.64 (dd, *J* = 13.1 and 2.9 Hz, 1H), 6.58 (dd, *J* = 9.2 and 2.9 Hz, 1H), 3.90 (q, *J* = 7.5 Hz, 1H), 3.74 (s, 3H), 3.25 (br, 1H), 2.98-2.96 (m, 1H), 2.18-2.14 (m, 1H), 2.08-2.02 (m, 2H), 1.70-1.59 (m, 2H), 1.33 (d, *J* = 7.5 Hz, 3H), 1.29-1.05 (m, 3H), 0.94-0.85 (m, 1H); ^13^C NMR (125 MHz, CDCl_3_) δ (ppm) 152.7 (d, ^1^*J*_(C, F)_ = 238 Hz), 151.8 (d, ^3^*J*_(C, F)_ = 9.5 Hz), 145.8, 130.2 (d, ^2^*J*_(C, F)_ = 11.9 Hz), 128.6, 126.8, 126.4, 114.8 (d, ^3^*J*_(C, F)_ = 3.6 Hz), 109.4 (d, ^4^*J*_(C, F)_ = 3.6 Hz), 102.4 (d, ^2^*J*_(C, F)_ = 22.7 Hz), 58.4, 57.9, 55.9, 54.3, 32.3, 31.3, 25.3, 24.9, 24.4; MS (FAB^+^) *m/z*: 342 (M^ +^, 100); HRMS (FAB^+^) *m/z*: calcd for C_21_H_27_FN_2_O (M^ +^): 342.2107. Found: 342.2109; [α]_D_^26^ = 50.7 (*c* 1.03, CHCl_3_). Single crystals suitable for X-ray diffraction were grown by cooling a solution of (1*R*, 2*R*, 1’*S*)-**4f** in hexane in a closed tube to -20 ºC. The crystal data of (1*R*, 2*R*, 1’*S*)-**4f** are as follows: space group, *P*2_1_; *a* = 8.5306(19) Å, *b* = 14.839(4) Å, *c* = 14.805(4) Å, *V* = 1874.1(8) Å^3^, *Z* = 4, *D_calc_* = 1.214 g/cm^3^, *R* = 0.0568, *R_w_* = 0.1334, GOF = 0.930. CCDC 768496 contains the supplementary crystallographic data for this paper. These data can be obtained free of charge from The Cambridge Crystallographic Data Centre via www.ccdc.cam.ac.uk/data_request/cif.

### 3.5. Synthesis of 1-(3,5-bis(trifluoromethyl)phenyl)-3-((1S,2S)-2-(2-fluoro-4-methoxyphenylamino)cyclohexyl)thiourea *[**(***S***, ***S***)-1f**]*

To a solution of (1*S*,2*S*,1’*S*)-**3f **(350 mg, 1.02 mmol) in CH_3_OH (15 mL) was added 10% Pd/C (100 mg) and ammonium formate (1.50 g). After the mixture was stirred at 60 °C for 12 h and cooled at ambient temperature, the mixture was filtered through through a pad of celite. The filtrate was concentrated *in vacuo*, The resulting material was dissolved with CH_2_Cl_2_ (5 mL), and added 1-isothiocyanato-3,5-bis(trifluoromethyl)benzene (280 mg, 1.00 mmol). After the mixture was stirred at ambient temperature for 3 h, the reaction mixture was concentrated in vacuo. The residue was purified by silica gel chromatography (hexane-ethyl acetate = 5:1) to give **(*S*, *S*)-1f **(436 mg, 0.856 mmol, 84%) as a colorless amorphous solid; IR (ATR) 3275, 2937, 1514, 1278, 1090 cm^−1^; ^1^H-NMR (500 MHz, DMSO-*d*_6_) δ (ppm) 9.98 (s, 1H), 8.27 (br, 1H), 8.17 (s, 2H), 7.69 (s, 1H), 6.78 (dd, *J* = 9.2 and 8.6 Hz, 1H), 6.72 (dd, *J* = 13.2 and 2.3 Hz, 1H), 6.61 (dd, *J* = 8.6 and 2.3 Hz, 1H), 4.69-4.66 (m, 1H), 4.39 (br, 1H), 3.65 (s, 3H), 3.32-3.26 (m, 1H), 2.21-2.02 (m, 2H), 1.74-1.67 (m, 2H), 1.41-1.20 (m, 4H); ^13^C-NMR (125 MHz, DMSO-*d*_6_) δ (ppm) 180.2, 150.4 (d, ^3^*J*_(C, F)_ = 9.5 Hz), 151.3 (d, ^1^*J*_(C, F)_ = 237 Hz), 141.9, 130.2 (q, ^2^*J*_(C, F)_ = 32.2 Hz), 129.8 (d, ^2^*J*_(C, F)_ = 11.9 Hz), 123.2 (q, ^1^*J*_(C, F)_ = 272 Hz), 121.8, 115.8, 113.5, 109.6, 102.1 (d, ^2^*J*_(C, F)_ = 22.6 Hz), 79.2, 56.7, 55.5, 31.9, 31.1, 24.4, 24.0; MS (FAB^+^) *m/z*: 438 (M + H^+^, 100); HRMS (FAB^+^) *m/z*: calcd for C_22_H_23_F_7_N_3_OS (M + H^+^): 510.1450. Found: 510.1438; [α]_D_^24^ = -49.0 (*c* 1.02, CHCl_3_).

### 3.6. Synthesis of* (1*S*,2*S*,1’*S*)**-3g*** and* (1*R*,2*R*,1’*S***)-4g***

By a similar procedure described for the preparation of (1*S*,2*S*,1’*S*)-**3f** and (1*R*,2*R*,1’*S*)-**4f**, (1*S*,2*S*, 1’*S*)-**3g** (550 mg, 1.55 mmol, 52%) and (1*R*,2*R*,1’*S*)-**4g** (270 mg, 0.76 mmol, 25%) were obtained from **2 **(603 mg, 3.00 mmol) and 2,4-dimethoxyaniline (470 mg, 3.07 mmol).

*(1S,2S)-N^1^-(2,4-Dimethoxyphenyl)-N^2^-((S)-1-phenylethyl)cyclohexane-1,2-diamine* [(1*S*,2*S*,1’*S*)-**3g**]: Colorless oil; IR (ATR) 3330, 2978, 2922 cm^−1^; ^1^H-NMR (500 MHz, CDCl_3_) δ (ppm) 7.34-7.18 (m, 5H), 6.64 (d, *J* = 8.6 Hz, 1H), 6.47 (d, *J* = 2.3 Hz, 1H), 6.40 (dd, *J* = 8.6 and 2.3 Hz, 1H), 3.89 (q, *J* = 6.9 Hz, 1H), 3.85 (s, 3H), 3.76 (s, 3H), 2.96 (dt, *J* = 3.6 and 9.9 Hz, 1H), 2.49 (dt, *J* = 3.8 and 9.7 Hz, 1H), 2.15-2.11 (m, 1H), 1.84-1.80 (m, 1H), 1.66-1.60 (m, 2H), 1.31 (d, *J* = 6.9 Hz, 3H), 1.28-1.00 (m, 4H); ^13^C-NMR (125 MHz, CDCl_3_) δ (ppm) 152.0, 148.7, 147.5, 132.5, 128.24, 128.23, 126.6, 112.1, 104.0, 99.3, 60.7, 58.7, 56.4, 55.8, 55.5, 32.8, 32.6, 24.9, 24.8, 24.2; MS (FAB^+^) *m/z*: 354 (M^+^, 100); HRMS (FAB^+^) *m/z*: calcd for C_22_H_30_N_2_O_2_ (M^ +^): 354.2307. Found: 354.2292; [α]_D_^26^ = 41.0 (*c* 0.98, CHCl_3_).

*(1R,2R)-N^1^-(2,4-Dimethoxyphenyl)-N^2^-((S)-1-phenylethyl)cyclohexane-1,2-diamine* [(1*R*,2*R*,1’*S*)-**4g**)]: Colorless oil; IR (ATR) 3335, 2974, 2926 cm^−1^; ^1^H-NMR (500 MHz, CDCl_3_) δ (ppm) 7.37-7.24 (m, 5H), 6.61 (d, *J* = 8.6 Hz, 1H), 6.46 (d, *J* = 2.9 Hz, 1H), 6.40 (dd, *J* = 8.6 and 2.9 Hz, 1H), 3.91 (q, *J* = 6.9 Hz, 1H), 3.85 (s, 3H), 3.76 (s, 3H), 3.05-2.98 (m, 1H)., 2.18-2.04 (m, 3H), 1.69-1.58 (m, 2H), 1.33 (d, *J* = 6.9 Hz, 3H), 1.24-1.05 (m, 3H), 0.91-0.82 (m, 1H); ^13^C-NMR (125 MHz, CDCl_3_) δ (ppm) 151.9, 148.60, 148.59, 132.1, 128.5, 126.7, 126.5, 111.8, 104.1, 99.3, 60.4, 58.1, 57.9, 55.8, 55.8, 32.2, 31.3, 25.4, 25.0, 24.5; MS (FAB^+^) *m/z*: 354 (M^+^, 100); HRMS (FAB^+^) *m/z*: calcd for C_22_H_30_N_2_O_2_ (M^+^): 354.2307. Found: 354.2291; [α]_D_^26^ = 20.8 (*c* 1.18, CHCl_3_).

### 3.7. Synthesis of 1-(3,5-bis(trifluoromethyl)phenyl)-3-((1S,2S)-2-(2,4-dimethoxyphenylamino)cyclo- hexyl)thiourea *[**(S, S)-1g**]*

By a similar procedure described for the preparation of **(*S*,*S*)-1f**, **(*S*,*S*)-1g** (502 mg, 0.96 mmol, 72%) was obtained from (1*S*,2*S*,1’*S*)-**3g (**470 mg, 1.33 mmol); Colorless amorphous Solid; IR (ATR) 3331, 2935, 1510, 1277, 1091 cm^−1^; ^1^H-NMR (500 MHz, DMSO-*d*_6_) δ (ppm) 9.94 (s, 1H), 8.25 (br, 1H), 8.18 (s, 2H), 7.70 (s, 1H), 6.55 (d, *J* = 8.6 Hz, 1H), 6.47 (d, *J* = 2.9 Hz, 1H), 6.37 (dd, *J* = 8.6 and 2.9 Hz, 1H), 4.47-4.31 (m, 2H), 3.71 (s, 3H), 3.65 (s, 3H), 3.31-3.14 (m, 1H), 2.20-2.01 (m, 2H), 1.77-1.61 (m, 2H), 1.46-1.06 (m, 4H); ^13^C-NMR (125 MHz, DMSO-*d*_6_) δ (ppm) 180.2, 150.9, 147.4, 141.9, 130.1 (q, ^2^*J*_(C, F)_ = 31.0 Hz), 123.2 (q, ^1^*J*_(C, F)_ = 272 Hz), 121.8, 115.8, 109.9, 104.3, 99.2, 56.6, 56.5, 55.4, 55.3, 31.9, 31.1, 31.2, 24.4, 24.0; MS (FAB^+^) *m/z*: 438 (M + H^+^, 100); HRMS (FAB^+^) *m/z*: calcd for C_23_H_26_F_6_N_3_O_2_S (M + H^+^): 522.1650. Found: 522.1633; [α]_D_^25^ = -14.5 (*c* 1.25, CHCl_3_).

### 3.8. Synthesis of* (1*S*,2*S*,1’*S*)**-3h*** and* (1*R*,2*R*,1’*S*)**-4h***

By a similar procedure described for the preparation of (1*S*,2*S*,1’*S*)-**3f** and (1*R*,2*R*,1’*S*)-**4f**, (1*S*,2*S*, 1’*S*)-**3h** (425 mg, 1.14 mmol, 57%) and (1*R*,2*R*,1’*S*)-**4h** (85 mg, 0.22 mmol, 11%) were obtained from **2 **(403 mg, 2.00 mmol) and 2-fluoro-4-isopropoxyaniline (340 mg, 2.00 mmol).

*(1S,2S)-N^1^-(2-Fluoro-4-isopropoxyphenyl)-N^2^-((S)-1-phenylethyl)cyclohexane-1,2-diamine* [(1*S*, 2*S*, 1’*S*)-**3h**]: Colorless oil; IR (ATR) 3335, 2974, 2926 cm^−1^; ^1^H-NMR (500 MHz, CDCl_3_) δ (ppm) 7.34-7.19 (m, 5H), 6.74 (dd, *J* = 9.1 and 8.6 Hz, 1H), 6.65 (dd, *J* = 13.2 and 2.9 Hz, 1H), 6.57 (dd, *J* = 8.6 and 2.9 Hz, 1H), 4.35 (seq, *J* = 6.3 Hz, 1H), 3.89 (q, *J* = 6.9 Hz, 1H), 3.69 (br, 1H), 2.93 (dt, *J* = 3.5 and 9.9 Hz, 1H), 2.47 (dt, *J* = 4.0 and 9.8 Hz, 1H), 2.14-2.10 (m, 1H), 1.87-1.83 (m, 1H), 1.67-1.61 (m, 2H), 1.31 (d, *J* = 6.9 Hz, 3H), 1.29 (d, *J* = 6.3 Hz, 6H), 1.28-1.00 (m, 4H); ^13^C-NMR (125 MHz, CDCl_3_) δ (ppm) 152.7 (d, ^1^*J*_(C, F)_ = 237 Hz), 149.8 (d, ^3^*J*_(C, F)_ = 9.5 Hz), 147.2, 130.5 (d, ^2^*J*_(C, F)_ = 12.1 Hz), 128.3, 126.6, 126.5, 114.9 (d, ^3^*J*_(C, F)_ = 4.8 Hz), 112.3 (d, ^4^*J*_(C, F)_ = 3.6 Hz), 104.7 (d, ^2^*J*_(C, F)_ = 20.7 Hz), 71.2, 60.5, 58.9, 56.2, 32.71, 32.66, 24.77, 24.74, 24.1, 22.1; MS (FAB^+^) *m/z*: 370 (M^+^, 100); HRMS (FAB^+^) *m/z*: calcd for C_23_H_31_FN_2_O (M^ +^): 370.2420. Found: 370.2414; [α]_D_^26^ = 30.0 (*c* 1.22, CHCl_3_).

*(1R,2R)-N1-(2-Fluoro-4-isopropoxyphenyl)-N2-((S)-1-phenylethyl)cyclohexane-1,2-diamine* [(1*R*,2*R*, 1’*S*)-**4h**]: Colorless oil; IR (ATR) 3361, 2977, 2923 cm^−1^; ^1^H-NMR (500 MHz, CDCl_3_) δ (ppm) 7.38-7.24 (m, 5H), 6.69 (dd, *J* = 9.8 and 8.6 Hz, 1H), 6.63 (dd, *J* = 12.6 and 2.9 Hz, 1H), 6.57 (dd, *J* = 9.8 and 2.9 Hz, 1H), 4.36 (seq, *J* = 6.3 Hz, 1H), 3.90 (q, *J* = 6.9 Hz, 1H), 3.29 (br, 1H), 2.99-2.95 (m, 1H)., 2.18-2.02 (m, 3H), 1.69-1.60 (m, 2H), 1.33 (d, *J* = 6.9 Hz, 3H), 1.29 (d, *J* = 6.3 Hz, 6H), 1.28-1.08 (m, 3H), 0.92-0.87 (m, 1H); ^13^C NMR (125 MHz, CDCl_3_) δ (ppm) 153.0 (d, ^1^*J*_(C, F)_ = 237 Hz), 149.7 (d, ^3^*J*_(C, F)_ = 9.5 Hz), 145.8, 130.4 (d, ^2^*J*_(C, F)_ = 11.9 Hz), 128.6, 126.9, 126.4, 114.7 (d, ^3^*J*_(C, F)_ = 4.8 Hz), 112.4 (d, ^4^*J*_(C, F)_ = 2.4 Hz), 104.8 (d, ^2^*J*_(C, F)_ = 22.7 Hz), 71.3, 58.4, 58.0, 54.3, 32.3, 31.3, 25.3, 24.9, 24.5, 22.1; HRMS (FAB^+^) *m/z*: calcd for C_23_H_31_FN_2_O (M^ +^): 370.2420. Found: 370.2411; [α]_D_^25^ = 43.7 (*c* 1.18, CHCl_3_).

### 3.9. Synthesis of 1-(3,5-bis(trifluoromethyl)phenyl)-3-((1S,2S)-2-(2-fluoro-4-isopropoxyphenylamino)cyclohexyl)thiourea* [**(***S***, ***S***)-1h**]*

By a similar procedure described for the preparation of **(*S*,*S*)-1f**, **(*S*,*S*)-1h** (410 mg, 0.762 mmol, 81%) was obtained from (1*S*,2*S*,1’*S*)-**3h **(350 mg, 0.944 mmol); Colorless amorphous Solid; IR (ATR) 3276, 2923, 1512, 1090 cm^−1^; ^1^H-NMR (500 MHz, DMSO-*d*_6_ at 100 °C) δ (ppm) 9.67 (s, 1H), 8.15 (s, 2H), 7.94 (br, 1H), 7.61 (s, 1H), 6.74 (dd, *J* = 9.2 and 9.2 Hz, 1H), 6.61 (d, *J* = 13.8 Hz, 1H), 6.55 (d, *J* = 9.2 Hz, 1H), 4.42-4.35 (m, 1H), 4.32 (seq, *J* = 5.8 Hz, 1H), 4.30 (br, 1H), 3.31-3.24 (m, 1H), 2.11-2.06 (m, 2H), 1.73-1.68 (m, 2H), 1.45-1.20 (m, 4H), 1.18 (d, *J* = 5.8 Hz, 6H); ^13^C-NMR (125 MHz, DMSO-*d*_6_ at 100 °C) δ (ppm) 180.3, 150.9 (d, ^1^*J*_(C, F)_ = 237 Hz), 148.4 (d, ^3^*J*_(C, F)_ = 9.5 Hz), 141.6, 129.8 (q, ^2^*J*_(C, F)_ = 33.4 Hz), 129.6 (d, ^2^*J*_(C, F)_ = 11.9 Hz), 122.7 (q, ^1^*J*_(C, F)_ = 271 Hz), 121.8, 115.4, 113.6 (d, ^3^*J*_(C, F)_ = 3.6 Hz), 112.2 (d, ^4^*J*_(C, F)_ = 3.6 Hz), 104.3 (d, ^2^*J*_(C, F)_ = 21.5 Hz), 70.4, 56.7, 56.6, 31.7, 30.7, 23.8, 23.5, 21.3; MS (FAB^+^) *m/z*: 438 (M + H^+^, 100); HRMS (FAB^+^) *m/z*: calcd for C_24_H_27_F_7_N_3_OS (M + H^+^): 538.1763. Found: 538.1760; [α]_D_^25^ = -48.3 (*c* 1.15, CHCl_3_).

### 3.10. Preparation of (Z)-ethyl 3-(4-methoxyphenylamino)acrylate *(**5a**)*

To a solution of ethyl 3-oxopropanoate (1.16 g, 10.0 mmol) in CH_2_Cl_2_ (20 mL) was added *p*-anisidine (1.23 g, 10.0 mmol) at ambient temperature. After the mixture was stirred at the same temperature overnight, the reaction mixture was concentrated. The resulting residue was passed through silica gel pad (hexane-ethyl acetate = 4:1) to afford the desired material as a *E/Z* mixture, which was recrystallized from hexane-ethyl acetate to give the title material **5a** (352 mg, 1.59 mmol, 16%, predominantly *Z* form) as a pale yellow solid; Mp. 48–49 °C (ethyl acetate-hexane); IR (ATR) 3276, 2981, 2918, 1699 cm^−1^; ^1^H-NMR (500 MHz, CDCl_3_) δ (ppm) 9.81 (brd, *J* = 12.6 Hz, 1H), 7.15 (dd, *J* = 12.6, 8.0 Hz, 1H), 6.91 (d, *J* = 8.6 Hz, 2H), 6.85 (d, *J* = 8.6 Hz, 2H), 4.77 (d, *J* = 8.0 Hz, 1H), 4.17 (q, *J* = 7.4 Hz, 2H), 3.78 (s, 3H), 1.30 (t, *J* = 7.4 Hz, 3H);^ 13^C-NMR (125 MHz, CDCl_3_) δ (ppm) 170.5, 155.5, 144.1, 134.5, 117.0, 114.9, 86.1, 59.1, 55.6, 14.5; MS (FAB^+^) *m/z*: 221 (M^+^, 100); HRMS (FAB^+^) *m/z*: calcd for C_12_H_15_NO_3_ (M^ +^): 221.1052. Found: 221.1069.

### 3.11. Preparation of enamino esters ***5b-k***

Enaminoesters **5b-k** were prepared using literature procedures [29]. Enaminoesters **5b-k** was used in the reactions without further purification.

### 3.12. General Procedure for the reaction of enaminoester ***5a*** with 4-nitrocinnamaldehyde *(**6a**)* catalyzed by thiourea ***1*** – Brønsted acid (Table 1 and Table 2).

To a solution of thiourea **1** (0.010 mmol) and Brønsted acid (0.010 mmol) in toluene (1.0 mL) were added **5a** (0.10 mmol) and **6a** (0.10 mmol) at ambient temperature. After being stirred at the same temperature, the reaction mixture was concentrated *in vacuo*. The resulting residue was purified by silica gel chromatography (hexane-ethyl acetate = 5:1) to give **7aa** and **8aa**.

*Ethyl 1-(4-methoxyphenyl)-6-(4-nitrophenyl)-1,6-dihydropyridine-3-carboxylate* (**8aa**): An orange oil; IR (ATR) 2979, 2919, 1685, 1513 cm^−1^; ^1^H-NMR (400 MHz, CDCl_3_) δ (ppm) 8.20 (d, *J* = 8.7 Hz, 2H), 7.80 (s, 1H), 7.49 (d, *J* = 8.7 Hz, 2H), 6.96 (d, *J* = 8.8 Hz, 2H), 6.82 (d, *J* = 8.8 Hz, 2H), 6.58 (d, *J* = 9.8 Hz, 1H), 5.66 (d, *J* = 5.4 Hz, 1H), 5.37 (dd, *J* = 9.8 and 5.4 Hz, 1H), 4.22 (q, *J* = 7.1 Hz, 2H), 3.77 (s, 3H), 1.30 (t, *J* = 7.1 Hz, 3H); ^13^C-NMR (125 MHz, CDCl_3_) δ (ppm) 166.2, 157.5, 149.5, 141.8, 138.1, 129.0, 126.4, 124.4, 122.2, 121.2, 114.7, 113.7, 102.3, 62.7, 59.8, 55.5, 14.5; MS (FAB^+^) *m/z*: 380 (M^+^, 100); HRMS (FAB^+^) *m/z*: calcd for C_21_H_20_N_2_O_5_ (M^ +^): 380.1372. Found: 380.1359.

### 3.13. Typical Procedure for the reaction of enaminoester ***5a*** with 4-nitrocinnamaldehyde ***6a*** catalyzed by thiourea ***(***S***, ***S***)-1h*** – difluoro acid (Table 3 and Table 4)

To a solution of **3a** (17.7 mg, 0.10 mmol) in toluene (0.40 mL) were added thiourea **(*S*, *S*)-1h** (5.4 mg, 0.010 mmol) and 0.1 M difluoroacetic acid in toluene solution (100 μL, 0.010 mmol) at ambient temperature. To this mixture was added dropwise (50 μL/30 min) a solution of **2a** (22.1 mg, 0.10 mmol) in toluene (0.50 mL) at ambient temperature. After being stirred at the same temperature for 12 h, the reaction mixture was concentrated *in vacuo*. The resulting residue was purified by silica gel chromatography (hexane-ethyl acetate = 5:1) to give **7aa** (32.7 mg, 86%) as a yellow oil.

*(R)-Ethyl 1-(4-methoxyphenyl)-4-(4-nitrophenyl)-1,4-dihydropyridine-3-carboxylate* (**7aa**): A yellow oil; IR (ATR) 2978, 2836, 1689, 1511 cm^−1^; ^1^H-NMR (400 MHz, CDCl_3_) δ (ppm) 8.18 (d, *J* = 8.8 Hz, 2H), 7.64 (d, *J* = 1.7 Hz, 1H), 7.52 (d, *J* = 8.8 Hz, 2H), 7.15 (d, *J* = 9.0 Hz, 2H), 6.94 (d, *J* = 9.0 Hz, 2H), 6.39 (dd, *J* = 8.0 and 1.7 Hz, 1H), 4.99 (dd, *J* = 8.0 and 4.6 Hz, 1H), 4.74 (d, *J* = 4.6 Hz, 1H), 4.14-4.00 (m, 2H), 3.83 (s, 3H), 1.15 (t, *J* = 7.1 Hz, 3H); ^13^C-NMR (125 MHz, CDCl_3_) δ (ppm) 167.4, 157.6, 154.6, 146.6, 138.7, 137.2, 128.7, 127.1, 123.7, 122.0, 114.9, 108.1, 103.4, 59.9, 55.6, 38.9, 14.3; MS (FAB^+^) *m/z*: 380 (M^+^, 100); HRMS (FAB^+^) *m/z*: calcd for C_21_H_20_N_2_O_5_ (M^ +^): 380.1372. Found: 380.1367; HPLC (CHIRALCEL AD-H, hexane-2-propanol = 80:20, flow rate 1.0 mL/min, 254 nm), t_r_(minor) = 18.1 min, t_r_(major) = 22.2 min. A sample with 50% ee by HPLC analysis gave [α]_D_^22^ = 125.2 (*c* 1.33, CHCl_3_).

*(R)-Ethyl 1-(4-chlorophenyl)-4-(4-nitrophenyl)-1,4-dihydropyridine-3-carboxylate* (**7ba**): By a similar procedure described for the preparation of **7aa**, **7ba** was obtained from **5b** and **6a** as a yellow oil (78%); IR (ATR) 2980, 2907, 1692, 1518 cm^−1^; ^1^H-NMR (400 MHz, CDCl_3_) δ (ppm) 8.18 (d, *J* = 8.8 Hz, 2H), 7.69 (d, *J* = 1.9 Hz, 1H), 7.50 (d, *J* = 8.8 Hz, 2H), 7.39 (d, *J* = 8.8 Hz, 2H), 7.15 (d, *J* = 8.8 Hz, 2H), 6.46 (dd, *J* = 7.8 and 1.9 Hz, 1H), 5.06 (dd, *J* = 7.8 and 4.6 Hz, 1H), 4.74 (d, *J* = 4.6 Hz, 1H), 4.15-4.05 (m, 2H), 1.16 (t, *J* = 7.1 Hz, 3H); ^13^C-NMR (125 MHz, CDCl_3_) δ (ppm) 167.1, 154.0, 146.6, 142.0, 137.4, 130.8, 129.9, 128.7, 126.0, 123.8, 121.0, 109.1, 105.0, 60.1, 38.8, 14.2; MS (FAB^+^) *m/z*: 384 (M^+^, 100); HRMS (FAB^+^) *m/z*: calcd for C_20_H_17_ClN_2_O_4_ (M^ +^): 384.0877. Found: 384.0887; HPLC (CHIRALCEL AD-H, hexane-2-propanol = 80:20, flow rate 1.0 mL/min, 254 nm), t_r_(minor) = 14.4 min, t_r_(major) = 19.5 min. A sample with 49% ee by HPLC analysis gave [α]_D_^25^ = 91.8 (*c* 1.09, CHCl_3_). 

*(R)-Ethyl 1-benzyl-4-(4-nitrophenyl)-1,4-dihydropyridine-3-carboxylate* (**7ca**): By a similar procedure described for the preparation of **7aa**, **7ca** was obtained from **5c** and **6a** as a yellow oil (83%); IR (ATR) 2979, 2922, 1683, 1516 cm^−1^; ^1^H-NMR (400 MHz, CDCl_3_) δ (ppm) 8.13 (d, *J* = 8.8 Hz, 2H), 7.43 (d, *J* = 8.8 Hz, 2H), 7.41-7.35 (m, 5H), 7.28 (s, 1H), 5.97 (d, *J* = 7.8 Hz, 1H), 4.86 (dd, *J* = 7.8 and 4.6 Hz, 1H), 4.68 (d, *J* = 4.6 Hz, 1H), 4.47 (s, 2H), 4.03 (m, 2H), 1.14 (t, *J* = 7.1 Hz, 3H); ^13^C- NMR (125 MHz, CDCl_3_) δ (ppm) 167.5, 154.9, 146.4, 140.8, 136.7, 129.0, 128.6, 128.2, 127.7, 127.1, 123.6, 107.5, 101.3, 59.7, 57.9, 38.7, 14.3; MS (FAB^+^) *m/z*: 365 (M + H^+^, 100); HRMS (FAB^+^) *m/z*: calcd for C_21_H_21_N_2_O_4_ (M + H^+^): 365.1501. Found: 365.1528; HPLC (CHIRALCEL AD-H, hexane/2-propanol = 85/15, flow rate 1.0 mL/min, 254 nm), t_r_(minor) = 14.9 min, t_r_(major) = 17.8 min. A sample with 45% ee by HPLC analysis gave [α]_D_^22^ = 117.6 (*c* 1.14, CHCl_3_).

*(R)-Ethyl 1-(4-methoxyphenyl)-2-methyl-4-(4-nitrophenyl)-1,4-dihydropyridine-3-carboxylate* (**7da**): By a similar procedure described for the preparation of **7aa**, **7da** was obtained from **5d** and **6a** as a yellow oil (93%); IR (ATR) 2979, 2918, 1685, 1512 cm^−1^; ^1^H-NMR (400 MHz, CDCl_3_) δ (ppm) 8.18 (d, *J* = 8.8 Hz, 2H), 7.50 (d, *J* = 8.8 Hz, 2H), 7.11 (d, *J* = 9.0 Hz, 2H), 6.94 (d, *J* = 9.0 Hz, 2H), 6.13 (d, *J* = 7.6 Hz, 1H), 4.93 (dd, *J* = 7.6 and 5.6 Hz, 1H), 4.81 (d, *J* = 5.6 Hz, 1H), 4.03 (q, *J* = 7.1 Hz, 2H), 3.84 (s, 3H), 2.16 (s, 3H), 1.12 (t, *J* = 7.1 Hz, 3H); ^13^C-NMR (125 MHz, CDCl_3_) δ (ppm) 168.2 158.9, 155.8, 149.6, 146.3, 136.1, 130.6, 128.8, 128.2, 123.7, 114.8, 105.7, 99.7, 59.6, 55.5, 40.5, 18.5, 14.3; MS (FAB^+^) *m/z*: 395 (M + H^+^, 100); HRMS (FAB^+^) *m/z*: calcd for C_22_H_23_N_2_O_5_ (M + H^+^): 395.1607. Found: 395.1607; HPLC (CHIRALCEL AD-H, hexane-2-propanol = 80:20, flow rate 1.0 mL/min, 254 nm), t_r_(minor) = 7.2 min, t_r_(major) = 16.7 min. A sample with 66% ee by HPLC analysis gave [α]_D_^23^ = 277.0 (*c* 1.20, CHCl_3_).

*(R)-tert-Butyl 1-(4-methoxyphenyl)-2-methyl-4-(4-nitrophenyl)-1,4-dihydropyridine-3-carboxylate* (**7ea**): By a similar procedure described for the preparation of **7aa**, **7ea** was obtained from **5e** and **6a** as a yellow oil (81%); IR (ATR) 2976, 2917, 1686, 1510 cm^−1^; ^1^H-NMR (400 MHz, CDCl_3_) δ (ppm) 8.19 (d, *J* = 8.8 Hz, 2H), 7.50 (d, *J* = 8.8 Hz, 2H), 7.10 (d, *J* = 8.6 Hz, 2H), 6.93 (d, *J* = 8.6 Hz, 2H), 6.09 (d, *J* = 7.6 Hz, 1H), 4.87 (dd, *J* = 7.6 and 5.4 Hz, 1H), 4.78 (d, *J* = 5.4 Hz, 1H), 3.83 (s, 3H), 2.13 (s, 3H), 1.30 (s, 9H); ^13^C-NMR (125 MHz, CDCl_3_) δ (ppm) 167.7, 158.8, 156.1, 148.6, 146.3, 136.3, 130.5, 128.8, 128.1, 123.7, 114.7, 105.4, 101.1, 79.5, 55.5, 41.0, 28.2, 18.4; MS (FAB^+^) *m/z*: 422 (M^+^, 100); HRMS (FAB^+^) *m/z*: calcd for C_24_H_26_N_2_O_5_ (M^+^): 422.1842. Found: 422.1852; HPLC (CHIRALCEL AD-H, hexane-2-propanol = 90:10, flow rate 0.5 mL/min, 254 nm), t_r_(minor) = 13.9 min, t_r_(major) = 18.5 min. A sample with 51% ee by HPLC analysis gave [α]_D_^23^ = 208.3 (*c* 1.30, CHCl_3_).

*(R)-Ethyl 1-(4-methoxyphenyl)-4-(4-nitrophenyl)-2-phenyl-1,4-dihydropyridine-3-carboxylate* (**7fa**): By a similar procedure described for the preparation of **7aa**, **7fa** was obtained from **5f** and **6a** as a yellow amorphous solid (85%); IR (ATR) 2980, 2917, 1674, 1511 cm^−1^; ^1^H-NMR (400 MHz, CDCl_3_) δ (ppm) 8.23 (d, *J* = 8.8 Hz, 2H), 7.67 (d, *J* = 8.8 Hz, 2H), 7.13-6.91 (m, 5H), 6.86 (d, *J* = 9.0 Hz, 2H), 6.64 (d, *J* = 9.0 Hz, 2H), 6.42 (d, *J* = 7.6 Hz, 1H), 5.08 (dd, *J* = 7.6 and 5.6 Hz, 1H), 4.88 (d, *J* = 5.6 Hz, 1H), 3.75-3.68 (m, 2H), 3.70 (s, 3H), 0.70 (t, *J* = 7.1 Hz, 3H); ^13^C-NMR (125 MHz, CDCl_3_) δ (ppm) 167.7, 157.7, 155.3, 150.9, 146.5, 136.1, 135.9, 131.2, 128.6, 128.3, 127.8, 127.41, 127.40, 123.9, 114.0, 106.1, 101.4, 59.5, 55.3, 40.3, 13.5; MS (FAB^+^) *m/z*: 457 (M + H^+^, 100); HRMS (FAB^+^) *m/z*: calcd for C_27_H_25_N_2_O_5_ (M + H^+^): 457.1763. Found: 457.1784; HPLC (CHIRALCEL AD-H, hexane-2-propanol = 93:7, flow rate 0.5 mL/min, 254 nm), t_r_(major) = 31.4 min, t_r_(minor) = 34.9 min. A sample with 61% ee by HPLC analysis gave [α]_D_^23^ = 237.0 (*c* 1.12, CHCl_3_).

*(R)-Ethyl 1-(3,4-dimethoxyphenyl)-2-methyl-4-(4-nitrophenyl)-1,4-dihydropyridine-3-carboxylate* (**7ga**): By a similar procedure described for the preparation of **7aa**, **7ga** was obtained from **5g** and **6a** as a yellow oil (96%); IR (ATR) 2977, 2933, 1687, 1511 cm^−1^; ^1^H-NMR (400 MHz, CDCl_3_) δ (ppm) 8.19 (d, *J* = 8.8 Hz, 2H), 7.51 (d, *J* = 8.8 Hz, 2H), 6.89 (d, *J* = 8.6 Hz, 1H), 6.76 (dd, *J* = 8.6 and 2.4 Hz, 1H), 6.67 (d, *J* = 2.4 Hz, 1H), 6.15 (d, *J* = 7.6 Hz, 1H), 4.93 (dd, *J* = 7.6 and 5.4 Hz, 1H), 4.83 (d, *J* = 5.4 Hz, 1H), 4.03 (q, *J* = 7.1 Hz, 2H), 3.92 (s, 3H), 3.90 (s, 3H), 2.18 (s, 3H), 1.12 (t, *J* = 7.1 Hz, 3H); ^13^C-NMR (125 MHz, CDCl_3_) δ (ppm) 168.2 155.8, 149.6, 149.5, 148.6, 146.3, 136.2, 130.5, 128.2, 123.7, 111.3, 105.6, 99.7, 59.6, 56.11, 56.07, 40.5, 18.5, 14.2; MS (FAB^+^) *m/z*: 425 (M + H^+^, 100); HRMS (FAB^+^) *m/z*: calcd for C_23_H_25_N_2_O_6_ (M + H^+^): 425.1713. Found: 425.1725; HPLC (CHIRALCEL AD-H, hexane-2-propanol = 80:20, flow rate 1.0 mL/min, 254 nm), t_r_(minor) = 10.2 min, t_r_(major) = 38.8 min. A sample with 66% ee by HPLC analysis gave [α]_D_^22^ = 239.5 (*c* 1.18, CHCl_3_).

*(R)-Ethyl 1-(3,4-dimethoxyphenyl)-2-methyl-4-phenyl-1,4-dihydropyridine-3-carboxylate* (**7gb**): By a similar procedure described for the preparation of **7aa**, **7gb** was obtained from **5g** and **6b** as a pale yellow oil (61%); IR (ATR) 2977, 2932, 1686, 1510 cm^−1^; ^1^H-NMR (400 MHz, CDCl_3_) δ (ppm) 7.38-7.29 (m, 5H), 7.21-7.16 (m, 1H), 6.87 (d, *J* = 8.6 Hz, 1H), 6.77 (dd, *J* = 8.6 and 2.4 Hz, 1H), 6.69 (d, *J* = 2.4 Hz, 1H), 6.12 (d, *J* = 7.8 Hz, 1H), 4.98 (dd, *J* = 7.8 and 5.4 Hz, 1H), 4.69 (d, *J* = 5.4 Hz, 1H), 4.02 (q, *J* = 7.1 Hz, 2H), 3.91 (s, 3H), 3.89 (s, 3H), 2.16 (s, 3H), 1.13 (t, *J* = 7.1 Hz, 3H); ^13^C-NMR (125 MHz, CDCl_3_) δ (ppm) 168.8, 149.5, 148.6, 148.35, 148.32, 136.7, 129.6, 128.3, 127.5, 126.1, 119.9, 111.3, 111.1 107.1, 101.1, 59.3, 56.06, 56.05, 40.2, 18.4, 14.2; MS (FAB^+^) *m/z*: 380 (M + H^+^, 100); HRMS (FAB^+^) *m/z*: calcd for C_23_H_26_NO_5_ (M + H^+^): 380.1862. Found: 380.1862; HPLC (CHIRALCEL AD-H, hexane-2-propanol = 80:20, flow rate 1.0 mL/min, 254 nm), t_r_(minor) = 6.1 min, t_r_(major) = 14.5 min. A sample with 44% ee by HPLC analysis gave [α]_D_^25^ = 154.0 (*c* 1.23, CHCl_3_).

*(R)-Ethyl 1-(3,4-dimethoxyphenyl)-4-(4-methoxyphenyl)-2-methyl-1,4-dihydropyridine-3-carboxylate* (**7gc**): By a similar procedure described for the preparation of **7aa**, **7gc** was obtained from **5g** and **6c** as a pale yellow oil (56%); IR (ATR) 2977, 2929, 1685, 1508 cm^−1^; ^1^H-NMR (400 MHz, CDCl_3_) δ (ppm) 7.28 (d, *J* = 8.8 Hz, 2H), 6.87 (d, *J* = 8.6 Hz, 1H), 6.86 (d, *J* = 8.8 Hz, 2H), 6.76 (dd, *J* = 8.6 and 2.4 Hz, 1H), 6.68 (d, *J* = 2.4 Hz, 1H), 6.12 (d, *J* = 7.6 Hz, 1H), 4.96 (dd, *J* = 7.6 and 5.4 Hz, 1H), 4.63 (d, *J* = 5.4 Hz, 1H), 4.03 (q, *J* = 7.1 Hz, 2H), 3.91 (s, 3H), 3.89 (s, 3H), 3.79 (s, 3H), 2.14 (s, 3H), 1.16 (t, *J* = 7.1 Hz, 3H); ^13^C-NMR (125 MHz, CDCl_3_) δ (ppm) 168.9, 158.0, 149.5, 148.3, 147.9, 141.1, 136.8, 129.5, 128.5, 119.9, 113.6, 111.3, 111.1 107.2, 101.5, 59.3, 56.07, 56.05, 55.2, 39.2, 18.4, 14.2; MS (FAB^+^) *m/z*: 409 (M^+^, 100); HRMS (FAB^+^) *m/z*: calcd for C_24_H_27_NO_5_ (M^ +^): 409.1889. Found: 409.1883; HPLC (CHIRALCEL AD-H, hexane-2-propanol = 80:20, flow rate 1.0 mL/min, 254 nm), t_r_(minor) = 7.6 min, t_r_(major) = 21.0 min. A sample with 38% ee by HPLC analysis gave [α]_D_^25^ = 136.1 (*c* 1.21, CHCl_3_).

*(R)-Ethyl 1-(3,4-dimethoxyphenyl)-4-(4-fluorophenyl)-2-methyl-1,4-dihydropyridine-3-carboxylate* (**7gd**): By a similar procedure described for the preparation of **7aa**, **7gd** was obtained from **5g** and **6d** as a pale yellow oil (62%); IR (ATR) 2981, 2935, 1685, 1507 cm^−1^; ^1^H-NMR (400 MHz, CDCl_3_) δ (ppm) 7.31 (dd, *J* = 8.8 and 5.9 Hz, 2H), 6.99 (dd, *J* = 8.8 and 8.8 Hz, 2H), 6.87 (d, *J* = 8.3 Hz, 1H), 6.76 (dd, *J* = 8.3 and 2.4 Hz, 1H), 6.67 (d, *J* = 2.4 Hz, 1H), 6.12 (d, *J* = 7.8 Hz, 1H), 4.95 (dd, *J* = 7.8 and 5.4 Hz, 1H), 4.68 (d, *J* = 5.4 Hz, 1H), 4.03 (q, *J* = 7.1 Hz, 2H), 3.91 (s, 3H), 3.89 (s, 3H), 2.15 (s, 3H), 1.14 (t, *J* = 7.1 Hz, 3H); ^13^C-NMR (125 MHz, CDCl_3_) δ (ppm) 168.7, 161.4 (d, ^1^*J*_(C, F)_ = 242 Hz), 149.5, 148.4, 148.3, 144.5 (d, ^4^*J*_(C, F)_ = 3.6 Hz), 136.6, 129.7, 128.9 (d, ^3^*J*_(C, F)_ = 8.4 Hz), 119.9, 114.9 (d, ^2^*J*_(C, F)_ = 21.4 Hz), 111.3, 111.0, 106.9, 101.1, 59.4, 56.09, 56.07, 39.5, 18.4, 14.2; MS (FAB^+^) *m/z*: 398 (M + H^+^, 100); HRMS (FAB^+^) *m/z*: calcd for C_23_H_25_FNO_4_ (M + H^+^): 398.1768. Found: 398.1751; HPLC (CHIRALCEL AD-H, hexane-2-propanol = 80:20, flow rate 1.0 mL/min, 254 nm), t_r_(minor) = 6.0 min, t_r_(major) = 13.1 min. A sample with 53% ee by HPLC analysis gave [α]_D_^27^ = 148.7 (*c* 1.73, CHCl_3_).

*(R)-Ethyl 1-(3,4-dimethoxyphenyl)-4-(3-fluorophenyl)-2-methyl-1,4-dihydropyridine-3-carboxylate* (**7ge**): By a similar procedure described for the preparation of **7aa**, **7ge** was obtained from **5g** and **6e** as a pale yellow oil (55%); IR (ATR) 2977, 2934, 1686, 1510 cm^−1^; ^1^H-NMR (400 MHz, CDCl_3_) δ (ppm) 7.28-7.23 (m, 1H), 7.13 (d, *J* = 7.6 Hz, 1H), 7.08-7.04 (m, 1H), 6.92-6.83 (m, 1H), 6.87 (d, *J* = 8.4 Hz, 1H), 6.76 (dd, *J* = 8.4 and 2.4 Hz, 1H), 6.68 (d, *J* = 2.4 Hz, 1H), 6.13 (d, *J* = 7.6 Hz, 1H), 4.96 (dd, *J* = 7.6 and 5.4 Hz, 1H), 4.70 (d, *J* = 5.4 Hz, 1H), 4.04 (q, *J* = 7.1 Hz, 2H), 3.91 (s, 3H), 3.89 (s, 3H), 2.16 (s, 3H), 1.14 (t, *J* = 7.1 Hz, 3H); ^13^C-NMR (125 MHz, CDCl_3_) δ (ppm) 168.6, 163.2 (d, ^1^*J*_(C, F)_ = 243 Hz), 151.3 (d, ^3^*J*_(C, F)_ = 6.0 Hz), 149.6, 148.7, 148.4, 136.5, 130.0, 129.5 (d, ^3^*J*_(C, F)_ = 8.4 Hz), 122.9 (d, ^4^*J*_(C, F)_ = 2.4 Hz), 119.9, 114.3 (d, ^2^*J*_(C, F)_ = 21.4 Hz), 112.9 (d, ^2^*J*_(C, F)_ = 21.4 Hz), 111.3, 111.0, 106.5, 100.6, 59.4, 56.08, 56.07, 40.0, 18.4, 14.2; MS (FAB^+^) *m/z*: 398 (M + H^+^, 100); HRMS (FAB^+^) *m/z*: calcd for C_23_H_25_FNO_4_ (M + H^+^): 398.1768. Found: 398.1748; HPLC (CHIRALCEL AD-H, hexane-2-propanol = 85:15, flow rate 1.0 mL/min, 254 nm), t_r_(minor) = 7.2 min, t_r_(major) = 13.0 min. A sample with 58% ee by HPLC analysis gave [α]_D_^26^ = 189.7 (*c* 1.35, CHCl_3_).

*(R)-Ethyl 1-(3,4-dimethoxyphenyl)-4-(2-fluorophenyl)-2-methyl-1,4-dihydropyridine-3-carboxylate* (**7gf**): By a similar procedure described for the preparation of **7aa**, **7gf** was obtained from **5g** and **6f** as a pale yellow oil (70%); IR (ATR) 2976, 2931, 1687, 1510 cm^−1^; ^1^H-NMR (400 MHz, CDCl_3_) δ (ppm) 7.39-7.34 (m, 1H), 7.19-7.09 (m, 2H), 7.02-6.97 (m, 1H), 6.87 (d, *J* = 8.6 Hz, 1H), 6.77 (dd, *J* = 8.6 and 2.4 Hz, 1H), 6.69 (d, *J* = 2.4 Hz, 1H), 6.05 (d, *J* = 7.6 Hz, 1H), 5.03 (d, *J* = 5.4 Hz, 1H), 4.97 (dd, *J* = 7.6 and 5.4 Hz, 1H), 3.99 (q, *J* = 7.1 Hz, 2H), 3.91 (s, 3H), 3.89 (s, 3H), 2.20 (s, 3H), 1.07 (t, *J* = 7.1 Hz, 3H); ^13^C-NMR (125 MHz, CDCl_3_) δ (ppm) 168.6, 159.6 (d, ^1^*J*_(C, F)_ = 244 Hz), 149.7, 149.5, 148.4, 136.6, 135.3 (d, ^2^*J*_(C, F)_ = 14.3 Hz), 130.0, 129.76, 129.71, 127.4 (d, ^3^*J*_(C, F)_ = 7.2 Hz), 124.1 (d, ^4^*J*_(C, F)_ = 3.6 Hz), 120.0, 115.0 (d, ^2^*J*_(C, F)_ = 22.6 Hz), 111.2 (d, ^3^*J*_(C, F)_ = 10.7 Hz), 105.7, 98.8, 59.3, 56.06, 56.04, 33.8, 18.3, 14.2; MS (FAB^+^) *m/z*: 398 (M + H^+^, 100); HRMS (FAB^+^) *m/z*: calcd for C_23_H_25_FNO_4_ (M + H^+^): 398.1768. Found: 398.1761; HPLC (CHIRALCEL AD-H, hexane-2-propanol = 80:20, flow rate 1.0 mL/min, 254 nm), t_r_(minor) = 6.0 min, t_r_(major) = 11.4 min. A sample with 44% ee by HPLC analysis gave [α]_D_^25^ = 175.8 (*c* 0.985, CHCl_3_).

*(R)-Ethyl 1-(4-chlorophenyl)-2-methyl-4-(4-nitrophenyl)-1,4-dihydropyridine-3-carboxylate* (**7ha**): By a similar procedure described for the preparation of **7aa**, **7ha** was obtained from **5h** and **6a** as a yellow oil (78%); IR (ATR) 2980, 2901, 1691, 1517 cm^−1^; ^1^H-NMR (400 MHz, CDCl_3_) δ (ppm) 8.18 (d, *J* = 8.8 Hz, 2H), 7.48 (d, *J* = 8.6 Hz, 2H), 7.42 (d, *J* = 8.8 Hz, 2H), 7.14 (d, *J* = 8.6 Hz, 2H), 6.15 (d, *J* = 7.6 Hz, 1H), 4.97 (dd, *J* = 7.6 and 5.4 Hz, 1H), 4.82 (d, *J* = 5.4 Hz, 1H), 4.03 (q, *J* = 7.1 Hz, 2H), 2.16 (s, 3H), 1.13 (t, *J* = 7.1 Hz, 3H); ^13^C-NMR (125 MHz, CDCl_3_) δ (ppm) 168.0, 155.3, 148.4, 146.4, 141.8, 133.5, 130.0, 129.9, 128.8, 128.2, 123.8, 106.3, 101.1, 59.7, 40.4, 18.6, 14.2; MS (FAB^+^) *m/z*: 398 (M^+^, 100); HRMS (FAB^+^) *m/z*: calcd for C_21_H_19_ClN_2_O_4_ (M^ +^): 398.1033. Found: 398.1052; HPLC (CHIRALCEL AD-H, hexane-2-propanol = 90:10, flow rate 1.0 mL/min, 254 nm), t_r_(minor) = 10.4 min, t_r_(major) = 23.9 min. A sample with 38% ee by HPLC analysis gave [α]_D_^25^ = 158.0 (*c* 1.22, CHCl_3_)..

*(R)-Ethyl 1-benzyl-2-methyl-4-(4-nitrophenyl)-1,4-dihydropyridine-3-carboxylate* (**7ia**): By a similar procedure described for the preparation of **7aa**, **7ia** was obtained from **5i** and **6a** as a yellow oil (81%); IR (ATR) 2979, 2925, 1684, 1516 cm^−1^; ^1^H-NMR (400 MHz, CDCl_3_) δ (ppm) 8.13 (d, *J* = 8.8 Hz, 2H), 7.39 (d, *J* = 8.8 Hz, 2H), 7.38-7.31 (m, 3H), 7.22-7.20 (m, 2H), 6.02 (d, *J* = 7.6 Hz, 1H), 4.93 (dd, *J* = 7.6 and 5.5 Hz, 1H), 4.78 (d, *J* = 5.5 Hz, 1H), 4.69 (d, *J* = 16.8 Hz, 1H), 4.59 (d, *J* = 16.8 Hz, 1H), 3.99 (q, *J* = 7.1 Hz, 2H), 2.46 (s, 3H), 1.09 (t, *J* = 7.1 Hz, 3H); ^13^C-NMR (125 MHz, CDCl_3_) δ (ppm) 168.3, 155.9, 149.8, 146.3, 137.6, 130.3, 129.0, 128.1, 127.7, 126.2, 123.6, 106.6, 99.3, 59.5, 53.8, 40.5, 16.0, 14.2; MS (FAB^+^) *m/z*: 378 (M^+^, 100); HRMS (FAB^+^) *m/z*: calcd for C_22_H_22_N_2_O_4_ (M^ +^): 378.1580. Found: 378.1578; HPLC (CHIRALCEL AD-H, hexane-2-propanol = 90:10, flow rate 1.0 mL/min, 254 nm), t_r_(minor) = 12.0 min, t_r_(major) = 15.3 min. A sample with 80% ee by HPLC analysis gave [α]_D_^23^ = 309.8 (*c* 1.36, CHCl_3_).

*(R)-Ethyl 1-(4-methoxybenzyl)-2-methyl-4-(4-nitrophenyl)-1,4-dihydropyridine-3-carboxylate* (**7ja**): By a similar procedure described for the preparation of **7aa**, **7ja** was obtained from **5j** and **6a** as a yellow oil (65%); IR (ATR) 2978, 2922, 1683, 1513 cm^−1^; ^1^H-NMR (400 MHz, CDCl_3_) δ (ppm) 8.12 (d, *J* = 8.1 Hz, 2H), 7.37 (d, *J* = 8.1 Hz, 2H), 7.13 (d, *J* = 8.6 Hz, 2H), 6.90 (d, *J* = 8.6 Hz, 2H), 6.01 (d, *J* = 7.5 Hz, 1H), 4.92 (dd, *J* = 7.5 and 5.7 Hz, 1H), 4.77 (d, *J* = 5.7 Hz, 1H), 4.62 (d, *J* = 16.6 Hz, 1H), 4.51 (d, *J* = 16.6 Hz, 1H), 3.99 (q, *J* = 7.1 Hz, 2H), 3.82 (s, 3H), 2.46 (s, 3H), 1.09 (t, *J* = 7.1 Hz, 3H); ^13^C-NMR (125 MHz, CDCl_3_) δ (ppm) 168.3, 159.2, 155.9, 149.9, 146.2, 130.3, 129.5, 128.1, 127.5, 123.6, 114.3, 106.5, 99.2, 59.5, 55.3, 53.3, 40.5, 16.0, 14.2; MS (FAB^+^) *m/z*: 408 (M^+^, 100); HRMS (FAB^+^) *m/z*: calcd for C_23_H_24_N_2_O_5_ (M^ +^): 408.1685. Found: 408.1701; HPLC (CHIRALCEL AD-H, hexane-2-propanol = 90:10, flow rate 1.0 mL/min, 254 nm), t_r_(minor) = 16.2 min, t_r_(major) = 19.3 min. A sample with77% ee by HPLC analysis gave [α]_D_^26^ = 286.6 (*c* 1.20, CHCl_3_).

### 3.14. General Procedure for the reaction of 4-nitro cinnamaldehyde ***6a*** and 4-methoxyaniline *(**9**)* with ethyl acetoacetate *(**10**)* catalyzed by thiourea ***1***—Brønsted acid (Table 5)

To a mixture of thiourea **1** (0.010 mmol ) and Brønsted acid (0.010 mmol) in toluene (1.0 mL) were added **6** (0.15 mmol) and **9** (0.10 mmol) at ambient temperature. After being stirred at the same temperature for 30 min, **10** (0.20 mmol) was then added, and the resulting mixture was stirred at the same temperature. The reaction mixture was concentrated *in vacuo*, and the resulting residue was purified by silica gel chromatography (hexane-ethyl acetate = 5:1) to give **7da**.

## 4. Conclusions

We have developed a Brønsted acid–bifunctional thiourea co-catalyzed asymmetric cycloaddition of β-enamino esters and α,β-unsaturated aldehydes to afford 1,3,4-trisubstituted and 1,2,3,4-tetrasubstituted 1,4-DHPs, which uses novel thiourea catalysts **1f** and **1h **as a source of chirality. With the use of different Brønsted acids such as DFA and HBF_4_ with the same bifunctional thiourea, both enantiomers of 4-substituted 1,4-dihydropyridine can be synthesized from the same starting materials. Both the Brønsted acid and bifunctional thiourea co-catalysts are important for determining the enantioselectivity and sense of chirality. 
